# Unraveling the anti-colorectal cancer mechanisms of *Acanthopanax senticosus* polysaccharide: a multi-omics investigation into gut microbiota-metabolism-immunity crosstalk

**DOI:** 10.3389/fphar.2026.1749532

**Published:** 2026-02-24

**Authors:** Jiaxin Jiang, Xiwu Zhang, Di Han, Qichao Liang, Le Yang, Ling Kong, Yu Guan, Hui Sun, Chang Liu, Ye Sun, Ying Han, Jie Zhang, Xijun Wang

**Affiliations:** 1 State Key Laboratory of Integration and Innovation of Classic Formula and Modern Chinese Medicine, National Chinmedomics Research Center, National TCM Key Laboratory of Serum Pharmacochemistry, Metabolomics Laboratory, Department of Pharmaceutical Analysis, Heilongjiang University of Chinese Medicine, Harbin, China; 2 State Key Laboratory of Dampness Syndrome, The Second Affiliated Hospital Guangzhou University of Chinese Medicine, Guangzhou, China; 3 Heilongjiang Wusulijiang Pharmaceutical Co., Ltd., Technology Innovation Center of Wusulijiang Ciwujia, Hulin, China

**Keywords:** 16S rRNA, *Acanthopanax senticosus* polysaccharides, colorectal cancer, metabolic reprogramming, TLR4/MyD88/NF-κB pathway

## Abstract

**Background:**

This study aimed to systematically investigate the anti-colorectal cancer efficacy and the underlying mechanisms of *Acanthopanax senticosus* polysaccharide with a focus on its role in modulating the gut microbiota-metabolism-immune axis.

**Methods:**

A homogeneous ASP fraction was structurally characterized using HPSEC, monosaccharide composition analysis, SEM, and FT-IR. Its anti-tumor activity was evaluated in a CT-26 tumor-bearing mouse model through histopathology, tumor inhibition rate, immune organ indices, and serum cytokine (IFN-γ, TNF-α, IL-2) assays. The potential mechanisms of action were elucidated by integrating 16S rDNA sequencing of gut microbiota, determination of short-chain fatty acids (SCFAs), untargeted serum metabolomics using UPLC-Q-TOF/MS, molecular docking studies, Western blot analysis of key signaling proteins, and validation through *in vitro* cell experiments.

**Results:**

ASP demonstrated significant dose-dependent anti-tumor activity, with the medium dose showing the highest efficacy (50.84% inhibition). It induced tumor cell apoptosis, normalized tumor-associated immune organ hypertrophy, and rebalanced pro- and anti-tumor cytokines. Metabolomics identified 12 key biomarkers, revealing that ASP primarily reversed CRC-induced disruptions in glycerophospholipid and tryptophan metabolism. Concurrently, ASP restored gut microbiota diversity, suppressed pro-inflammatory genera, and promoted beneficial bacteria and some SCFAs. Integrated correlation analysis established a robust link between microbiota remodeling and metabolic correction. Molecular docking, Western blot validation and *vitro* cell experiments confirmed that ASP and its regulated metabolites could inhibit the activity of PLA2/TLR4/MyD88/NF-κB signaling pathway.

**Conclusion:**

The results indicate that anti-CRC effect of ASP may be jointly regulated through multiple pathways: correcting abnormal glycerophospholipid and tryptophan metabolism in the host, restoring the homeostasis of the intestinal microbiota, increase the content of some SCFAs, and inhibiting the TLR4/NF-κB signaling pathway.

## 1 Introduction

*Acanthopanax senticosus* (AS), a prominent medicinal plant within the Araliaceae family, has been extensively utilized in traditional Asian medicine for its remarkable immunomodulatory properties (Lau et al., 2019; Shi et al., 2024). Phytochemical investigations have identified multiple bioactive constituents in AS, including polysaccharides, flavonoids, and saponins, which collectively contribute to its diverse pharmacological activities (Xiong et al., 2013; Hu et al., 2015; Hu et al., 2018). Among these, *A. senticosus* polysaccharide (ASP) stands out due to its favorable safety profile and broad spectrum of biological effects, including antioxidant (Fu et al., 2012), anti-inflammatory (Liu A. et al., 2023), antiviral, immunomodulatory, and anti-tumor activities (Sun et al., 2019), positioning it as a promising natural therapeutic candidate for cancer treatment. Preliminary studies have demonstrated that ASP exhibits significant anti-tumor efficacy in murine models of S180 sarcoma, H22 hepatocellular carcinoma, and U14 cervical carcinoma (Meng et al., 2018). However, the specific effects and underlying mechanisms of ASP in colorectal cancer (CRC), particularly in CT-26 cell-induced models, remain inadequately explored.

Globally, CRC represents the third most prevalent cancer diagnosis and the second leading cause of cancer-related mortality, constituting a substantial healthcare burden worldwide. This malignancy is characterized by high invasiveness and metastatic potential, yet current therapeutic modalities face significant limitations in both efficacy and tolerability (Fillon, 2023; Pan et al., 2023). While surgical resection remains the primary clinical intervention, its success is frequently compromised by tumor metastasis and recurrence, necessitating adjuvant therapies that often carry substantial adverse effects (Singh et al., 2024). Although multimodal approaches combining surgery, chemotherapy, and radiotherapy continue to serve as the cornerstone of CRC management, these strategies frequently result in severe systemic toxicity, damage to healthy tissues, and incomplete disease control. These limitations underscore the urgent need for novel treatment options with improved safety profiles and enhanced therapeutic efficacy (Liang et al., 2025).

The development of such novel therapies requires a paradigm shift from conventional single-target approaches to comprehensive multi-mechanism strategies. Natural polysaccharides like ASP represent particularly promising candidates due to their inherent capacity to simultaneously modulate multiple biological pathways. Preliminary evidence from our laboratory has indicated that ASP can significantly inhibit tumor growth in CT-26 cell-induced CRC mouse models. Nevertheless, the high heterogeneity of CRC pathogenesis complicates the elucidation of ASP’s mechanism of action, as conventional single-dimensional experimental approaches fail to capture its potential multi-pathway synergistic effects. Critical knowledge gaps persist regarding how ASP remodels the gut microbiota and corrects host metabolic dysregulation in the CRC microenvironment, and the key molecular pathways through which ASP exerts its immunomodulatory and anti-tumor effects remain to be fully characterized. Most importantly, the potential mechanistic connections between microbiota remodeling, metabolic reprogramming, and inflammatory pathway regulation in ASP-mediated CRC suppression have not been systematically investigated. To address these fundamental questions, our study employs an integrated multi-omics strategy to decipher ASP’s anti-CRC underlying mechanisms, which combined 16S rDNA sequencing of gut microbiota with untargeted serum metabolomics using UPLC-Q-TOF/MS technology to characterize ASP-induced alterations in the microbial community and host metabolism. Furthermore, the research integrated molecular docking simulations and Western blot validation to identify the critical signaling pathways involved. The integrated regulatory axis of “microbiota - metabolism - inflammation” was proposed and systematically elucidated. The data indicate that ASP may ameliorate CRC by modulating the gut microbiota, regulating glycerophospholipid and tryptophan metabolism, and influencing the TLR4/MyD88/NF-κB signaling pathway. This systematic research strategy not only provides potential mechanistic insights for the clinical application of ASP but also establishes a novel methodological framework for investigating the effects of complex natural products on multifactorial diseases.

## 2 Materials and methods

### 2.1 Chemicals and reagents

The AS herbal medicine was provided by Ussurijiang Pharmaceutical Co., Ltd. in (Heilongjiang, China), any of the plant components used in the study do not derive from protected or endangered species. CT-26 colon carcinoma cells were obtained from provided by Beina Biotechnology Co., LTD. (Hebei, China). Fetal bovine serum (FBS) was obtained from Shanghai Dianrui Biotechnology Co., Ltd. (Shanghai, China). 5-fluorouracil (5-FU) and hematoxylin and eosin (HE) staining solution were supplied by Shanghai yuanye Bio-Technology Co., Ltd. (Shanghai, China). Enzyme-linked immunosorbent assay kits for IFN-γ (H025-1–2), IL-2 (H003-1–2), and TNF-α (H052-1–2) were purchased from Nanjing Jiancheng Institute of Bioengineering (Hangzhou, China). Total protein extraction kit, BCA protein assay kit, PLA2 (05–143), TLR4 (AF8187), MYD88 (AF2116), NF-κB (AN365) and glyceraldehyde-3-phosphate dehydrogenase (marker, GAPDH, AG019) primary antibody, goat anti rabbit secondary antibody (A0208) and ECL (P0018S) were purchased from Shanghai Biyun Tian Biotechnology Co., Ltd. (Shanghai, China). FlexA-200 microplate reader (Hangzhou, China). The above experimental supplies were funded by the Department of Science and Technology of Heilongjiang Province. LC-MS grade methanol and acetonitrile were provided by Fisher Scientific (Loughborough, United Kingdom). IX71 Inverted fluorescence microscope provided by Olympus Corporation (Japan). The identification of AS herbal medicine was conducted by Professor Wang Xijun, a professor in the Department of Pharmacognosy at Heilongjiang University of Chinese Medicine.

### 2.2 Extraction and purification of ASP

The medicinal herb underwent washing, drying, crushing, and sieving to obtain the powder. The powder was extracted with double-distilled water at a 1:20 ratio via refluxing twice, each time for 3 h. After filtration and combining the filtrates, the solution was concentrated under reduced pressure at 60 °C. Ethanol was then added to the concentrate under vigorous agitation, followed by allowing the mixture to stand undisturbed at room temperature overnight to induce polysaccharide precipitation. The precipitated polysaccharides were isolated through centrifugation, washed three times with ethanol, and finally dried at 60 °C, yielding the ASP crude extract. Deproteinization and decolorization were performed using D941 macroporous weakly basic anion exchange resin, after which dialysis and lyophilization were employed to obtain the ASP lyophilized powder. The crude extraction rate of ASP obtained by this method is 2.26%, and the yield of the refined product is approximately 20% (compared with the crude product), that is, 100 g of medicinal materials can eventually produce about 0.452 g of refined products. Total sugar content, determined by the phenol-sulfuric acid assay, was measured at 87.66% ± 0.84%.

### 2.3 Structure and chemical characterization of ASP

HPSEC chromatography was used to evaluate the Mw of ASP (Xu et al., 2014). The analysis system is Agilent 1,260, equipped with two TM250 and TM500 Waters Ultrahydrogel™ linear columns (7.8 × 300 mm, United States), combine with Agilent refractive index detector (RID, G1362A) for measurement. Wash with 0.1 mol/L NaNO3, flow rate of 1 mL/min. The temperature of the chromatographic column and RID is 40 °C, running time is 30 min. ASP was hydrolyzed by TFA, then its monosaccharide composition was determined by with PMP pre-column derivatization coupled to HPLC (Wang et al., 2014). The chromatographic column was Dikma Technologies Diamonsil C18 column (4.6 × 200 mm, 5 μm, China). Six monosaccharide standards encompassing mannose (Man), rhamnose (Rha), galacturonic acid (GalA), glucose (Glc), galactose (Gal), and arabinose (Ara) as references. The SEM (Zeiss Sigma 500, Germany) was utilized to observe the surface morphology of ASP. At different magnifications, the acceleration voltage is 5.0 kV under vacuum. The FT-IR spectrum of ASP was obtained by compressing 2 mg ASP sample into particles using KBr method and using Nicolet iS5 FT-IR spectrometer (Thermo Fisher Scientific, United States) in the range of 4000-400 cm^-1^ (Liu X. et al., 2023).

### 2.4 CT-26 cells induced CRC mouse model and experimental design

Male BALB/c mice (22 ± 2 g) were purchased from Liaoning Changsheng Biotechnology Co., Ltd. (SCXK 2020–0001) and housed under standard conditions. After 1 week of acclimatization, CT-26 colon carcinoma cells (5 × 10^6^ cells in 0.2 mL PBS) were subcutaneously injected into the right flank of each mouse. Tumor-bearing mice were randomly divided into five groups (n = 8): model (M), ASP low- (ASPL, 50 mg/kg), medium- (ASPM, 100 mg/kg), and high-dose (ASPH, 200 mg/kg), and positive control (5-FU, 25 mg/kg). Treatments were administered orally (ASP) or intraperitoneally (5-FU) for 21 days. Non-tumor-bearing mice served as normal controls (C). Tumor volume was measured every 3 days and calculated as V = 0.5 × L × W^2^. At the end of the experiment, blood, tumor tissues, thymus, spleen, and cecal contents were collected for further analysis. All procedures were approved by the Ethics Committee of Heilongjiang University of Chinese Medicine (No. 2023121507).

### 2.5 Histopathology, cytokine, and immune organ index analysis

Tumor tissues were fixed, embedded, sectioned, and stained with hematoxylin and eosin (H&E) for histopathological evaluation. Serum levels of IFN-γ, TNF-α, and IL-2 were measured using commercial ELISA kits according to the manufacturer’s instructions. The immune organ index was calculated as organ weight (mg)/body weight (g) × 100%. The tumor inhibition rate (TIR) was determined as: TIR (%) = (W_M_ − W_T_)/W_M_ × 100%, where W_M_ and W_T_ denote the mean tumor weights of the M and treatment groups, respectively.

### 2.6 Untargeted metabolomics analysis

Blood was collected from mice by removing the eyeballs. Serum samples were mixed with four volumes of methanol, vortexed, and centrifuged at 13,000 rpm for 15 min at 4 °C. The supernatant was filtered and analyzed using a UPLC-QTOF-MS system (Waters Corporation). Chromatographic separation was performed on an ACQUITY UPLC HSS T3 column (100 mm × 2.1 mm, 1.8 μm) with a gradient of 0.1% formic acid in water (A) and acetonitrile (B). The gradient elution: 0–2 min, 1%–40% A; 2–3 min, 40%–60% A; 3–8.5 min, 60%–100% A; 8.5–12 min, 100%–1% A. Mass spectrometry was conducted in both positive and negative ionization modes. Data were processed using Progenesis QI and MassLynx V4.1. Multivariate analyses including PCA and OPLS-DA were applied to identify differential metabolites. Potential biomarkers were annotated via HMDB and KEGG databases, and metabolic pathway analysis was performed using MetaboAnalyst 5.0.

### 2.7 The gut microbiota of CRC mice was analyzed by 16S rDNA technology

Cecal contents from each mouse group were collected into sterile cryotubes, rapidly frozen in liquid nitrogen, and stored at −80 °C for subsequent use. Total genomic DNA was extracted from samples, with concentration and purity assessed via NanoDrop spectroscopy and agarose gel electrophoresis. The V3-V4 hypervariable regions of the 16S rRNA gene were amplified using barcode-labeled specific primers and high-fidelity polymerases. Amplification products were purified and sequenced on an Illumina NovaSeq 6,000 platform. For data analysis, cutadapt was first applied to trim adapter sequences from raw FASTQ files. DADA2 was then used for quality filtering, denoising, sequence concatenation, and chimera removal, yielding an amplicon sequence variant (ASV) table and its representative sequences. QIIME 2 software annotated these representative sequences against reference databases for species identification. Subsequent analyses included α and β diversity assessments and intergroup differential species identification via the LEfSe method.

### 2.8 The effect of APS on SCFAs in the feces of CT-26 tumor-bearing mice

The procedure was performed following established literature methods. Approximately 70 mg of mouse intestinal content was homogenized with 400 μL of sodium chloride solution and 20 μL of 10% H_2_SO_4_ solution by vortexing for 3 min. Subsequently, 800 μL of diethyl ether was added for extraction, followed by ultrasonication at 4 °C for 10 min. After centrifugationfor 5 min, the ether layer was collected and dried over anhydrous sodium sulfate to remove residual moisture. The extract was then filtered through a 0.22 μm membrane into a brown glass vial for subsequent analysis.

Short-chain fatty acids (SCFAs) were quantified using gas chromatography-mass spectrometry (GC-MS). Separation was achieved on an HP-INNOWax capillary column (30 m × 0.25 mm × 0.25 μm; Agilent, United States) under a constant helium carrier gas flow (purity >99.99%) of 1.0 mL/min. The oven temperature program was set as follows: initial hold at 60 °C for 1 min, increased to 150 °C at 10 °C/min, then raised to 240 °C at 40 °C/min, and finally held at 240 °C for 3 min. MS detection was conducted in EI mode at 70 eV, with the ion source and transfer line temperatures maintained at 230 °C and 280 °C, respectively.

### 2.9 Molecular docking

Numerous studies indicates that lipid molecules could bind to TLR4, a pivotal immune receptor, triggering downstream signaling (Pal et al., 2012; Lancaster et al., 2018; Anggara et al., 2023). In this study, key phospholipid compounds LysoPC(22:6), PC(17:0/0:0), PC(37:6), and LysoPC(20:4/0:0) were selected for molecular docking with three target proteins TLR4, MyD88, and NF-κB. The 3D structures of potential active compounds were retrieved from PubChem (https://pubchem.ncbi.nlm.nih.gov/), while protein targets were acquired from the RCSB Protein Data Bank (https://www.rcsb.org/). Using AutoDockTools for preprocessing, receptor-ligand docking was performed to evaluate binding affinities, with results visualized through PyMOL.

### 2.10 The inhibitory effect of L-tryptophan on CT-26 cells

The inhibitory effect of L-tryptophan (a key metabolite of ASP) on CT-26 cells was evaluated by the CCK-8 method. Experimental setting with control group and experimental group: CT-26 cells in the control group were cultured in complete medium; The experimental groups were treated with L-tryptophan at concentrations of 6.25,12.5, 25, 50 and 100 µM respectively, with 6 duplicate wells set for each concentration. After culturing for 12, 24 and 48 h respectively, 10 μL of CCK-8 solution was added to each well and incubated at 37 °C for 2 h. Subsequently, the absorbance (OD value) at a wave length of 450 nm was detected using the FlexA-200 microplate reader. The formula for calculating the cell inhibition rate is: inhibition rate (%) = [1 - (average OD value of the experimental group/average OD value of the control group)] × 100%. All experiments were independently repeated three times to ensure the reliability of the results.

### 2.11 Western blot analysis

Tumor specimens and CT-26 cells were homogenized in a protein extraction buffer to prepare total protein lysates. The concentration of proteins was determined using the BCA assay. Proteins were separated by SDS-PAGE and subsequently transferred onto PVDF membranes. The membranes were blocked with 5% non-fat milk at room temperature for 1 h with gentle agitation, followed by overnight incubation at 4 °C with primary antibodies against TLR4, MyD88, and NF-κB. After a 60-min incubation at room temperature with a goat anti-rabbit IgG secondary antibody, protein bands were detected via ECL chemiluminescence and imaged using a Bio-Rad system. Finally, band intensities were quantified using ImageJ software.

### 2.12 Statistical analysis

The experimental data are presented as the mean ± standard deviation (SD). Statistical evaluations were conducted employing one-way ANOVA followed by Duncan’s multiple range test for identifying statistical disparities. The major experimental results were analyzed and processed with GraphPad Prism (GraphPad Software Inc., San Diego, CA, United States). Statistical significance was determined based on p-values, p < 0.05 indicates a significant difference in the data, p < 0.01 indicates an extremely significant difference in the data. ^*^p < 0.05 versus C group; ^**^p < 0.01 versus C group; ^#^p < 0.05 versus M group; ^##^p < 0.01 versus M group.

## 3 Results

### 3.1 Structural characterization reveals ASP as a well-defined acidic heteropolysaccharide

**FIGURE 1:**
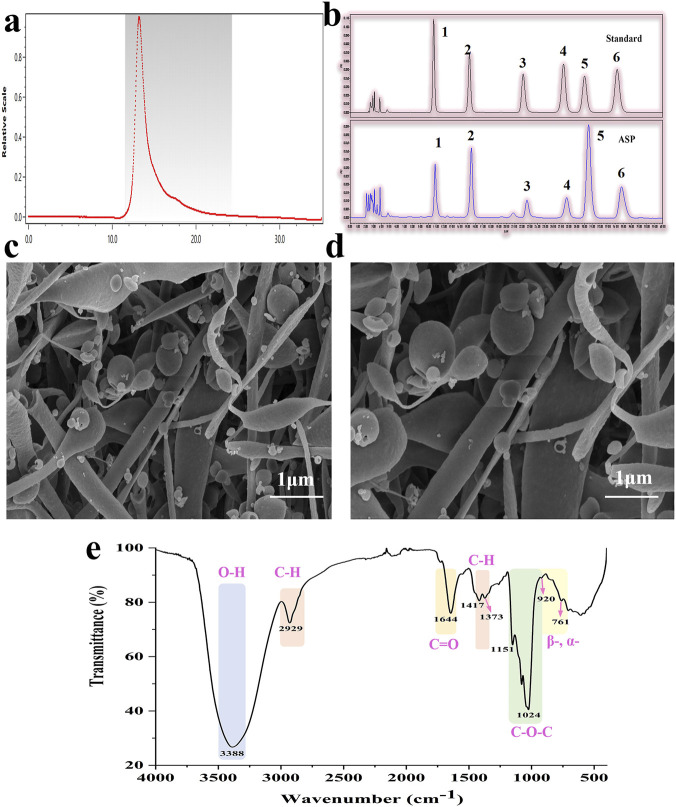
Fundamental structural characterization of ASP. **(a)** Molecular weight (Mw) of ASP: Obtained HPSEC-IR spectra with sharp and evenly distributed ASP peaks; **(b)** Monosaccharide compositions of ASP: Composed of 6 monosaccharides, they are 1: Mannose (Man), 2: Rhamnose (Rh), 3: Galacturonic acid (GalA), 4: Glucose (Glc), 5: Galactose (Gal), 6: Arabinose (Ara); **(c,d)** Scanning electron microscopy (SEM) image of ASP: Observe the surface and internal structure under.

This study begins by characterizing the basic structural properties of ASP, as these attributes constitute the basis for its biological functions. High-performance size exclusion chromatography (HPSEC) analysis revealed a homogeneous profile characterized by a single, symmetrical peak (Figure 1a), corresponding to a weight-average molecular weight (Mw) of 55.15 kDa. Monosaccharide composition analysis further identified ASP as an acidic heteropolysaccharide consisting of six monosaccharides: Man, Rha, GalA, Glc, Gal, Ara, with a molar ratio of 1.52:1:1.64:3.09:3.90:2.05 (Figure 1b). Notably, Glc (23.41%) and Gal (29.55%) collectively constituted over half of the total content, suggesting their potential role as backbone components, while the significant presence of GalA (12.42%) substantiated the acidic nature of this polymer, which could be crucial for its charge-dependent biological interactions. Scanning electron microscopy (SEM) analysis revealed an irregular, amorphous network structure composed of smooth spherical particles and rod-shaped formations with internal pores (Figures 1c,d). This highly porous and heterogeneous architecture implies a substantial specific surface area, potentially facilitating water retention, enzyme accessibility, and bioactive compound embedding. Furthermore, the observation of numerous small spherical particles adherent to larger aggregates suggests complex aggregation behavior during preparation or natural assembly. Fourier transform-infrared (FT-IR) spectroscopic analysis provided deeper insights into the functional groups and glycosidic linkage patterns (Figure 1e). The spectrum confirmed extensive hydroxyl groups (O-H stretching vibration at ∼3,388 cm^-1^) and aliphatic C-H bonds (vibrations at ∼2,929, 1,417, and 1,373 cm^-1^) (Peng et al., 2024). Critically, the strong C=O stretching vibration at 1,644 cm^-1^ not only confirmed the presence of uronic acid but also indicated capacity for hydrogen bonding and ionic interactions. The fingerprint region provided conclusive evidence for the pyranose form of constituent sugars (C-O-C stretches at 1,151 and 1,024 cm^-1^) and, most significantly, the coexistence of both α- and β-glycosidic linkages (characteristic peaks at ∼920 and 761 cm^-1^) (Shi et al., 2020). This heterotypic linkage configuration indicates that ASP is not composed by a single system, suggesting intricate biosynthetic pathways. These complexities likely account for the observed structural heterogeneity and may facilitate diverse biological functions through interactions with multiple cellular receptors.

### 3.2 ASP demonstrates significant anti-tumor efficacy through tumor suppression and immunomodulation

**FIGURE 2:**
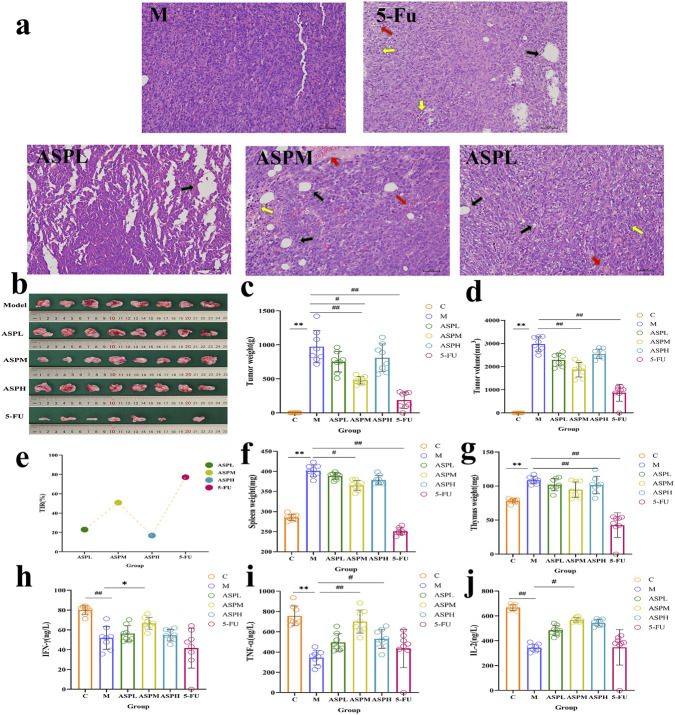
The regulatory effect of ASP intervention on the classical pathological indicators of CT-26 tumor-bearing mice. **(a)** Histopathological evaluation of tumor tissues in CT-26 tumor-bearing mice by Hematoxylin and Eosin (HE) staining (×200 magnification, scale bar: 50 μm. All sections were evaluated by two independent pathologists blinded to treatment groups). **(b–e)** Photographs of tumor tissues in CRC mice at treatment completion with different administration groups and effects of oral ASP on tumor weight, tumor volume, tumor inhibition rate (TIR), immune organ index (IOI), and biochemical indicators in CRC mice. **(f–j)**: Effects of different administration groups on immune organs of spleen and thymus weight and levels of IFN-γ, TNF-α and IL-2 in CRC mice. (n = 8; compared with control group **(c)**, * represents significant relevance (p < 0.05), ** represents extremely significant relevance (p < 0.01); compared with M group (M), # represents significant relevance (p < 0.05), ## represents extremely significant relevance (p < 0.01), mean ± SD).

To systematically evaluate the anti-tumor potential of ASP, the study first assessed its impact on tumor histoarchitecture through histopathological examination. Hematoxylin and eosin (H&E) staining revealed that tumor cells in the M group displayed tight, orderly arrangements with plump nuclei and intact morphology, consistent with active proliferation. In contrast, both ASP-treated and 5-FU-treated groups exhibited characteristic features of apoptosis, including disrupted cell-cell junctions, nuclear pyknosis, and increased intercellular gaps. Furthermore, ASP treatment induced significant vacuolization, inflammatory infiltration, and necrosis, leading to blurred cellular structures and severely disrupted tissue morphology. Notably, these pathological alterations were most prominent in the medium-dose ASP group (Figure 2a), suggesting a potent, dose-dependent capacity of ASP to induce tumor cell death and dismantle tumor integrity. We next quantified the anti-tumor potency of ASP by monitoring tumor progression. During model induction, tumor weight and volume in the M group increased exponentially. Post-treatment, both ASP and the positive control 5-FU significantly suppressed this growth, though ASP exhibited lower efficacy than 5-FU (Figures 2b–d). The tumor inhibition rates were calculated as 22.97% (ASPL), 50.84% (ASPM), and 16.83% (ASPH), compared to 77.12% for 5-FU (Figure 2e). The superior efficacy of the medium dose indicates a non-monotonic, potentially biphasic therapeutic window for ASP, a phenomenon frequently observed with biological response modifiers (Beers et al., 2018).

Given that the tumor organs often induce systemic immune dysfunction, we investigated whether ASP’s efficacy involved immunomodulation. The thymus and spleen indices, recognized indicators of immune status, were significantly elevated in the M group compared to C group, reflecting tumor-associated immune organ hypertrophy and dysfunction. ASP treatment significantly reversed this trend, reducing the spleen index by 17.35% (from 20.08 to 16.60) and the thymus index by 15.53% (from 5.10 to 4.31) (Figures 2f,g). This normalization suggests that ASP alleviates tumor-driven excessive immune activation and restores immune homeostasis, whereas 5-FU, despite its potent anti-tumor action, caused severe immunosuppression. Then, we analyzed serum levels of key anti-tumor cytokines. The M group displayed markedly suppressed IFN-γ levels, consistent with an immunosuppressed state. ASP treatment, particularly in the ASPM group, significantly restored IFN-γ production (increased by 15.65%; Figure 2h), implying that ASP may activate the Th1-type immune response crucial for anti-tumor immunity. Conversely, the M group showed elevated levels of IL-2 and TNF-α compared to C group, indicative of a dysregulated cytokine network that can paradoxically exacerbate immunosuppression. ASP treatment effectively normalized these levels, increasing IL-2 by 28.86% and TNF-α by 30.32% compared to the M group (Figures 2i,j). Overall, the results suggest that ASP may inhibit tumor growth by regulating the levels of immune factors (Reem and Yeh, 1984; Karki et al., 2020; Chen et al., 2021).

### 3.3 Metabolomics results indicated ASP-mediated correction of glycerophospholipid and tryptophan metabolic pathways

**FIGURE 3:**
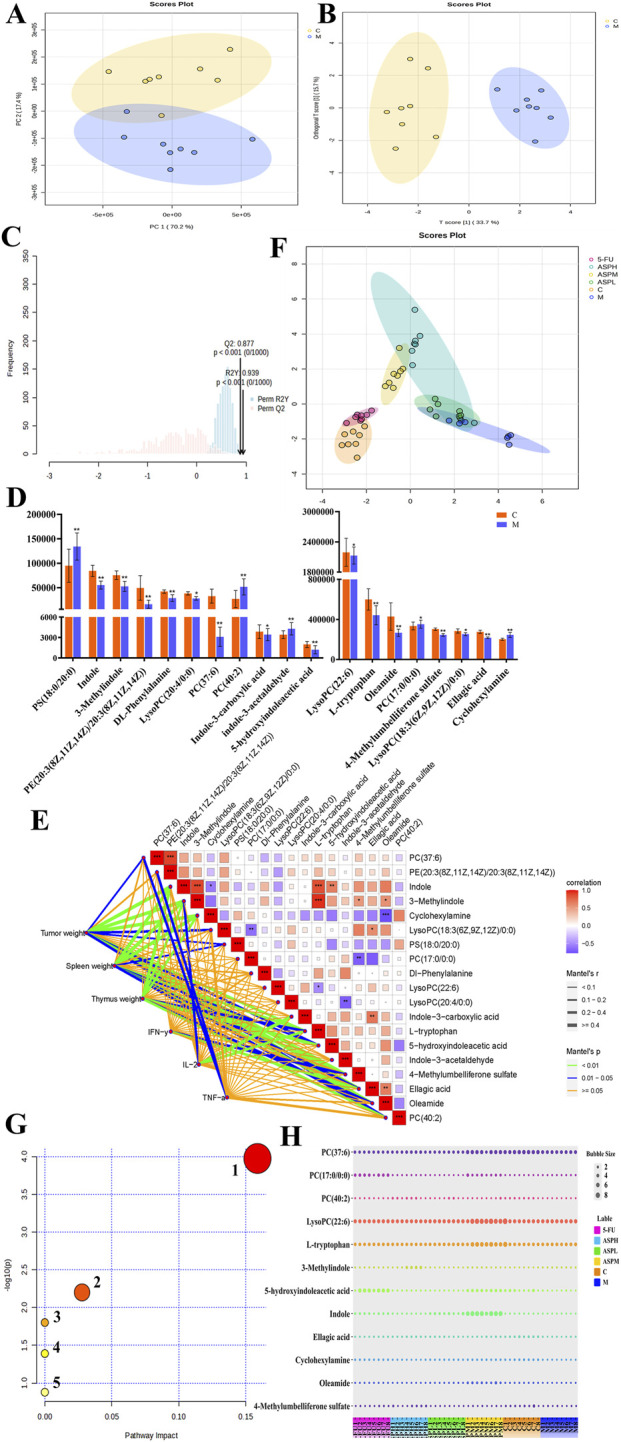
Metabolomics data analysis of CRC model mice after ASP intervention. **(A,B)** PCA and OPLS-DA evaluation of blood metabolic profile in groups C and M mice (groups C and M, n = 8); **(C)** Evaluation of stable and reliable parameters of OPLS-DA model: Q^2^ is 0.877 (greater than 0.5), implying the model is reliable. R^2^Y is 0.939 (close to 1), demonstrating model has strong explanatory power and predictability; **(D)** The 19 biomarkers relative content in CRC mice; **(E)** Correlation analysis between blood biomarkers and phenotypic data. **(F)** PCA score plots after ASP treatment (six groups, n = 8); **(G)** Biomarkers relative content in CRC mice after ASP treatment; **(H)** Metabolic profiling altered by ASP intervention in CRC mice (1. Glycerophospholipid metabolism; 2. Tryptophan metabolism; 3. Linoleic acid metabolism; 4. α-Linolenic acid metabolism; 5. Arachidonic acid metabolism).

To delineate the metabolic alterations underlying CRC and the intervention mechanism of ASP, we conducted an untargeted metabolomics study. Serum samples from C and M groups were analyzed via positive/negative ion scanning, generating base peak intensity (BPI) chromatograms that demonstrated high data quality (Supplementary Figure S1). Multivariate analysis revealed clear separation between the C and M groups in both principal component analysis (PCA) and orthogonal projections to latent structures-discriminant analysis (OPLS-DA) score plots (Figures 3A,B), indicating profound perturbations in the systemic metabolism of tumor-bearing mice. The robustness of the OPLS-DA model was validated through permutation testing, which confirmed a low risk of overfitting (Figure 3C). We subsequently identified 19 potential biomarkers through MS/MS fragmentation combined with HMDB and METLIN database matching (Supplementary Table S1). These are Dl-Phenylalanine, 5-hydroxyindoleacetic acid, indole, 3-Methylindole, L-tryptophan, indole-3-carboxylic acid, cyclohexylamine, indole-3-acetaldehyde, 4-Methylumbelliferone sulfate, ellagic acid, LysoPC(22:6), LysoPC(18:3 (6Z,9Z,12Z)/0:0), LysoPC(20:4/0:0)PC(37:6), PC(17:0/0:0), PS(18:0/20:0), PE (20:3 (8Z,11Z,14Z)/20:3 (8Z,11Z,14Z)), oleamide, PC(40:2), mainly consists of indole and phospholipid compounds. Correlation analysis between these metabolites and key clinical parameters (including tumor weight, immune organ indices, and cytokine levels) refined this list to 12 pivotal biomarkers (Figures 3D,E), including PC(37:6), LysoPC(22:6), L-tryptophan, and 5-hydroxyindoleacetic acid, etc. The significant dysregulation of these low-molecular-weight metabolites points to a systemic disruption of metabolic homeostasis.

So, the metabolic trajectory shifts associated with ASP treatment and disease phenotype. The PCA score plot demonstrated that oral administration of ASP, particularly at the medium dose, effectively shifted the global metabolic profile of tumor-bearing mice away from the M group and toward the healthy C cluster, a restorative effect paralleled in the 5-FU group (Figure 3F). This metabolic reconstitution was quantified through bubble plot visualization of biomarker abundance (Figure 3G). Among the 12 key biomarkers, ASPM treatment significantly reversed the aberrant levels of 7 metabolites, primarily encompassing glycerophospholipids (LysoPC(22:6), PC(17:0/0:0), PC(37:6), LysoPC(20:4/0:0)) and tryptophan metabolites (indole, L-tryptophan, 5-hydroxyindoleacetic acid). The collective metabolic changes were mapped onto a global network to elucidate the targeted pathways (Figure 3H). It mainly involves six metabolic pathways: glycerophospholipid metabolism, tryptophan metabolism, linoleic acid metabolism, α-linolenic acid metabolism, GPI-anchor biosynthesis, arachidonic acid metabolism. Pathway impact analysis identified glycerophospholipid metabolism as the primary pathway through which ASP exerts its anti-CRC effects, with tryptophan metabolism ranking second (Supplementary Table S2). The coordinated reversal of specific lysophosphatidylcholines and phosphatidylcholines indicates that ASP ameliorates CRC-driven phospholipid membrane remodeling and restores lipid homeostasis. Concurrently, the normalization of key tryptophan metabolites suggests that ASP alleviates tumor-associated inflammation and immune dysregulation via the kynurenine/indole pathway. In conclusion, the anti-tumor efficacy of ASP is accompanied by multi-faceted metabolic alterations, characterized by a shift in glycerophospholipid and tryptophan metabolic profiles towards those observed in healthy controls.

### 3.4 ASP restores gut microbiota homeostasis by reshaping community structure and composition

**FIGURE 4:**
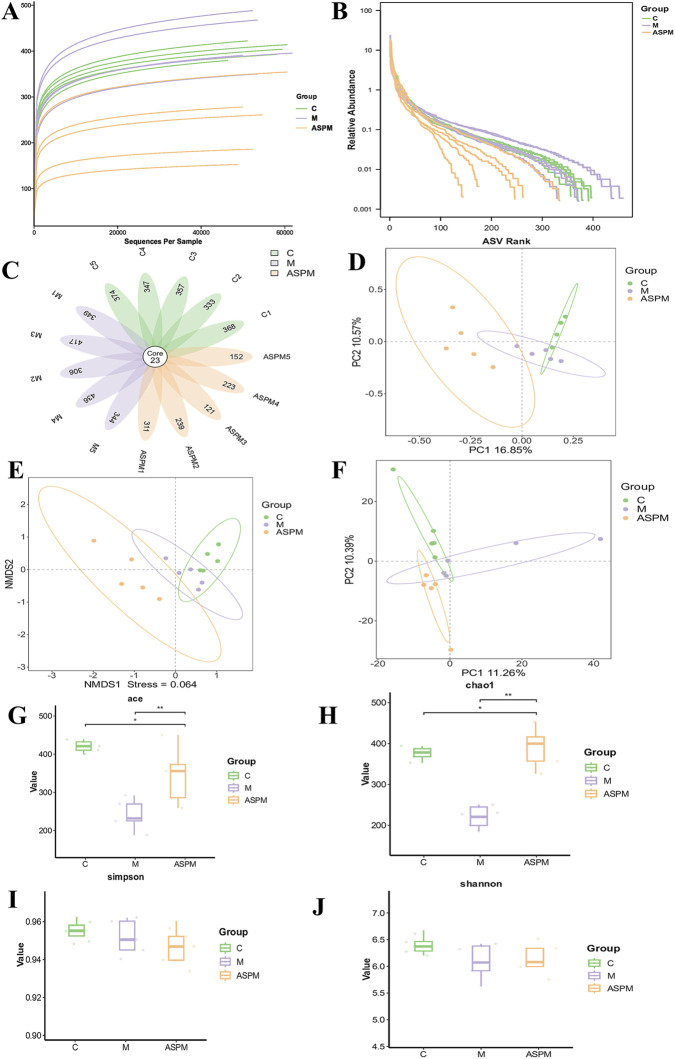
Availability analysis of gut microbiota data. **(A)** Dilution curve; **(B)** Rank abundance curve; **(C)** Petal diagram; **(D–F)** β diversity analysis: PCoA, NMDS and PCA analysis; **(G–J)** α diversity analysis: ace index, chao, shannon and simpson index.

Given the established role of intestinal microbiota dysbiosis in CRC, we employed 16S rDNA sequencing to investigate whether ASP’s anti-tumor effect is mediated through microbial regulation. Prior to analysis, data quality was assessed through dilution and rank-abundance curves, which confirmed sufficient sequencing depth and reliable species distribution (Figures 4A,B). Profiling of Amplicon Sequence Variants (ASVs) revealed a stark reduction in microbial richness in the M group (1,046 ASVs) compared to the C group (1852 ASVs). Crucially, ASP intervention largely restored this richness (1779 ASVs), and the shared ASV analysis further indicated that ASP treatment shifted the microbial composition toward a state resembling healthy controls (Figure 4C). This suggests that ASP effectively counteracts CRC-driven microbial depletion. The structural shifts in the microbial community were quantitatively assessed through β-diversity analysis. Non-metric multidimensional scaling, principal coordinate analysis, and PCA all demonstrated clear separation between the C and M groups, evidencing CRC-induced dysbiosis. Notably, the ASPM group clustered distinctly from the M group and converged toward the C group profile (Figures 4D–F), indicating a systemic restoration of gut microbiota structure by ASP. This restructuring was further corroborated by α-diversity analysis. The Ace and Chao indices, which reflect species richness, were significantly lower in the M group but were markedly recovered by ASPM treatment (Figures 4G,H), demonstrating ASP’s capacity to rebuild a diverse microbial ecosystem. In contrast, the Shannon and Simpson indices, indicative of community evenness, remained stable across groups (Figures 4I,J), implying that ASP’s primary restorative effect targets the restoration of lost microbial species rather than altering the dominance hierarchy (Zhu et al., 2022).

**FIGURE 5:**
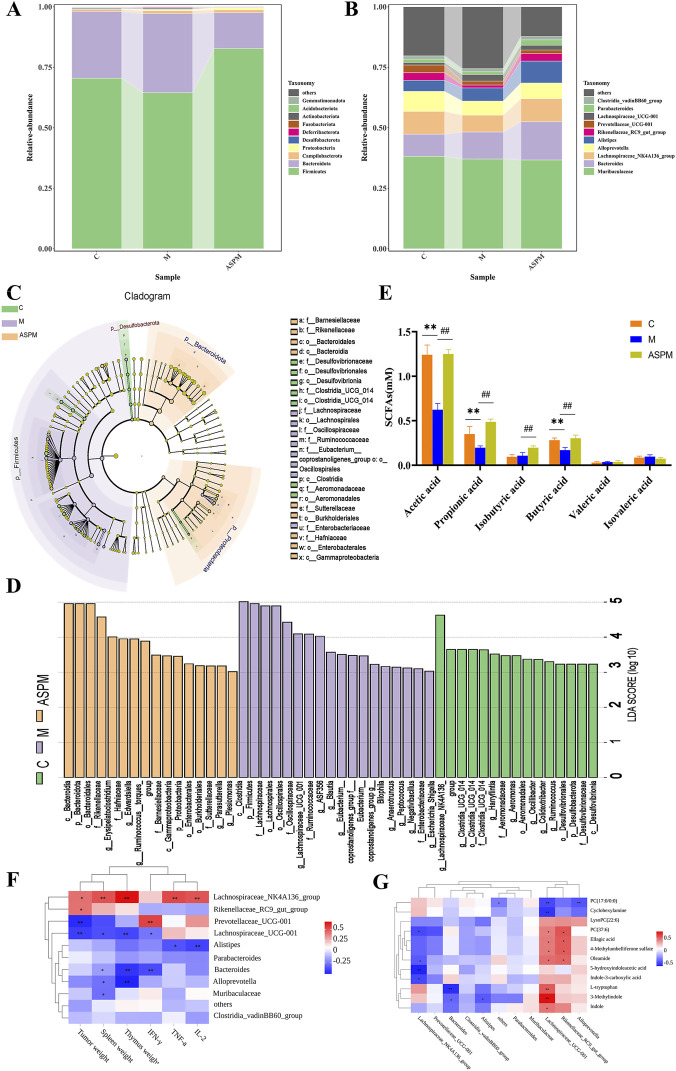
Analysis of the group results of the regulatory effect of ASP on the intestinal flora of mice with CRC. **(A)** Bar chart of the composition of the intestinal microbiota at phylum level; **(B)** Bar chart of the composition of the intestinal microbiota at genus level; **(C)** LEfSe analysis of differential species score plots; **(D)** LEfSe analysis of differential species annotation branch example diagram; **(E)** Changes in SCFAs content; **(F)** Spearman correlation analysis of gut microbiota and pathophysiological indicators; **(G)** Spearman correlation analysis of gut microbiota and key biomarkers (n = 5, * represents significant relevance (p < 0.05), ** represents extremely significant relevance (p < 0.01), mean ± SD).

To identify specific microbial populations modulated by ASP, we analyzed community composition at the phylum level. The M group exhibited a significant reduction in Firmicutes, a cornerstone phylum for maintaining intestinal homeostasis, alongside increases in Bacteroidota and Campylobacterota. This imbalance led to a marked elevation in the Bacteroidota-to-Firmicutes (B/F) ratio, a recognized hallmark of dysbiosis. ASP intervention effectively counteracted these changes, restoring Firmicutes abundance and lowering the B/F ratio (Figure 5A), which likely contributes to reduced chronic inflammation and a less procarcinogenic environment. A more granular analysis at the genus level provided potential mechanistic insights into ASP’s anti-tumor activity. The M group was characterized by proliferation of pro-inflammatory genera such as *Bacteroides* and Lachnospiraceae_UCG-001, alongside a decline in beneficial genera like Lachnospiraceae_NK4A136_group. ASP treatment reversed this dysbiotic pattern by significantly suppressing the pathobionts and restoring beneficial bacteria abundance (Figure 5B). To definitively identify discriminative microbial taxa, we performed linear discriminant analysis effect size (LEfSe) analysis (LDA >4). The results revealed distinct enriched taxa in each group (Figures 5C,D). The C group was characterized by beneficial genera including g_Lachnospiraceae_NK4A136_group and g_Clostridia_UCG_014, known for maintaining epithelial barrier integrity and suppressing inflammation. The M group showed enrichment of a broad range of taxa within the Firmicutes phylum, including c_Clostridia and f_Lachnospiraceae, reflecting a disease-associated dysbiotic state. Most importantly, the ASPM group exhibited a distinct enrichment pattern characterized by c_Bacteroidia, p_Bacteroidota, o_Bacteroidales, and f_Rikenellaceae. This specific signature indicates that ASP does not merely reverse the microbiota to a pre-disease state but actively promotes a unique, beneficial structural reorganization of the microbial community.

### 3.5 APS significantly increases the content of SCFAs, a key metabolite of the microbiota, maintaining intestinal homeostasis balance

Above research indicates that APS markedly influences the structural composition of the intestinal microbial community (Xu et al., 2024). The SCFAs act as critical mediators through which the microbiota modulates host immune homeostasis and colon health (Parada Venegas et al., 2019), this study further quantified the levels of major SCFAs in fecal samples from each experimental group (Figure 5E). SCFA metabolic profiling revealed that, compared with group C, the concentrations of acetic, propionic, and butyric acids were significantly reduced in group M (p < 0.05), consistent with the characteristic metabolic alterations observed in CRC (Parada Venegas et al., 2019). Following ASPM intervention, the levels of these three SCFAs were notably restored to baseline (p > 0.05), suggesting that ASP may alleviate CRC-associated pathological changes by modulating intestinal metabolite concentrations and thereby correcting microbial dysbiosis.

Furthermore, no significant intergroup differences were observed in the levels of valeric acid and isovaleric acid, indicating that ASPM exerts limited regulatory effects on the metabolism of these SCFAs (Liu et al., 2021). Notably, the concentration of isobutyric acid in the ASPM group was significantly higher than that in both the C and M groups (p < 0.05), implying that ASPM may contribute to intestinal repair by enhancing the synthesis or absorption of isobutyric acid. Collectively, these findings showed that ASP attenuates CRC progression by restoring physiological levels of key SCFAs-particularly acetic, propionic, and butyric acids-which in turn may exert their beneficial effects by enhancing intestinal barrier integrity, promoting immune regulation, and rebalancing the gut microbial ecosystem (Liu et al., 2021).

### 3.6 Integrated analysis reveals ASP acts through microbiota-metabolism-inflammation-TLR4/NF-κB axis

To systematically elucidate the anti-CRC mechanism of ASP, we performed an integrated analysis correlating gut microbiota, key serum metabolites, and pathophysiological indicators. Spearman correlation analysis revealed a complex tripartite interaction network linking these three components (Figure 5F). Key pathological indicators, including tumor weight and immune organ indices, showed significant positive correlations with pro-inflammatory cytokines (IFN-γ, TNF-α, IL-2) and the beneficial bacterial genus Lachnospiraceae_NK4A136_group. Conversely, these clinical parameters were negatively correlated with other genera, such as *Bacteroides* and Lachnospiraceae_UCG-001. Furthermore, correlation analysis between microbiota and metabolic biomarkers indicated that the identified metabolites were positively correlated with Lachnospiraceae_UCG-001 but negatively correlated with Lachnospiraceae_NK4A136_group (Figure 5G). These results suggest that ASP’s therapeutic effect is achieved may through rectifying gut microbiota dysbiosis, which in turn orchestrates a downstream recalibration of host metabolism and immune-inflammatory responses.

**FIGURE 6:**
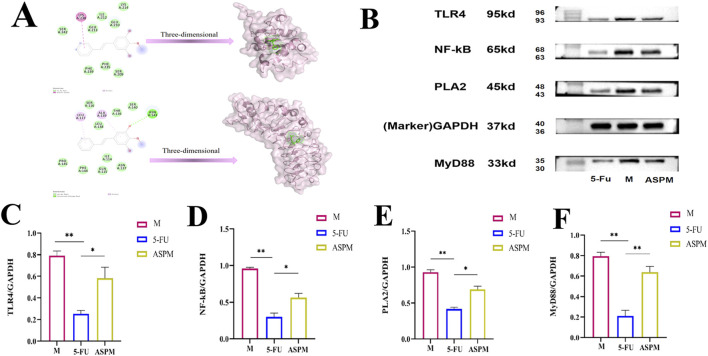
Metabolites docking with TLR4 pathway proteins and effects of ASP on the expression of TLR4/MyD88/NF-κB signaling proteins in CRC mice tumor tissues. **(A)** Visualization results of LysoPC(22:6) docking with TLR4 and MyD88 protein; **(B)** The Western blot test results of PLA2, TLR4, MyD88, and NF-κB in tumor issues of mice in different treatment groups: The expression levels of four proteins in ASPM group were significantly lower than in M group, slightly higher than in 5-FU group; **(C–F)** Evaluation of treatment group variations in protein expression: Quantify four proteins expression in figure **(B)** (Compared with group M, * representatives significant relevance (p < 0.05), ** representatives extremely significant relevance (p < 0.01, mean ± SD).

**FIGURE 7:**
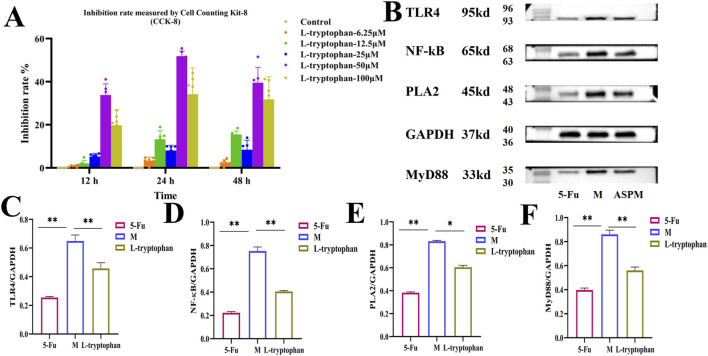
The effect of L-tryptophan on the inhibition rate of CT-26 cells and the TLR4/MyD88/NF-κB signaling pathway. **(A)** After culturing CT-26 cells with different concentrations of L-tryptophan medium, the cell inhibition rate: With the increase of drug concentration and the extension of action time, the cell inhibition rate increased. The inhibition effect was the best when the concentration was 50 μM and the cells were cultured for 24 h. It was 51.67%; **(B)** Western blot detection results of PLA2, TLR4, MyD88 and NF-κB proteins in CRC of different groups: The expression levels of the four proteins in the L-tryptophan group were significantly lower than those in the M group and slightly higher than those in the 5-FU group; **(C–F)** Changes in protein expression in different groups: Quantify the expression levels of the four proteins in Figure B (compared with group M, * represents a significant correlation (p < 0.05), ** represents an extremely significant correlation (p < 0.01, mean ± SD).

To delineate potential targets and underlying molecular events, we investigated interactions between ASP metabolites and key receptors in innate immune signaling using molecular docking. Simulations predicted potential high-affinity binding of ASP’s bioactive metabolites and the target proteins (TLR4, MyD88, and NF-κB), evidenced by negative binding energies indicative of spontaneous and stable complex formation (Figure 6A; Supplementary Table S3). The stability of these complexes was driven by extensive hydrophobic contacts and precise hydrogen bond networks within the protein’s binding pocket. This predicted binding suggests that ASP metabolites may be associated with the modulation of the TLR4 signaling pathway, which could contribute to the rectification of metabolic and inflammatory dysregulation observed in CRC progression. It should be noted that molecular docking provides predictive insights rather than experimental evidence of direct binding. Finally, to experimentally validate pathway involvement, we assessed protein expression of key signaling molecules in tumor tissues via Western blot analysis. Results demonstrated that ASPM treatment, akin to the positive control 5-FU, significantly modulated expression of proteins in the TLR4 pathway (Figure 6B). Specifically, ASPM administration led to marked downregulation of PLA2, TLR4, MyD88, and NF-kB protein expression compared to the M group, although the effect was less pronounced than with 5-FU (Figures 6C–F). In addition, the results of *in vitro* cell experiments indicated that ASP’s metabolite L-tryptophan had a certain inhibitory effect on CRC cells. When the administration dose was 50 μM and the culture lasted for 24 h *in vitro*, the inhibition rate of CRC reached highest to 51.67%. The results of Western blot further verified that L-tryptophan has a certain regulatory effect on the TLR4/MyD88/NF-kB pathway (Figure 7). In conclusion, these data describe the multi-faceted responses to ASP treatment: the restoration of intestinal microbiota homeostasis, the increase of beneficial SCFAs, and the correction of metabolic disorders combined. *In vitro* experiments have shown that L-tryptophan can inhibit the growth of CRC cells.

## 4 Discussion

This multidisciplinary study integrated chemical analysis, histopathology, immunology, metabolomics, microbiome sequencing, and molecular biology to systematically elucidate the anti- CRC efficacy and the multi-dimensional underlying mechanisms of ASP (Zhang et al., 2020). Starting with the precise structural characterization of ASP, we progressively uncovered a complete mechanistic cascade: ASP could modulate host immunity, restore gut microbiota homeostasis, reverse disruptions in key metabolic pathways, and ultimately suppress the activation of the PLA2/TLR4/MyD88/NF-κB signaling axis (Yin et al., 2022). This work not only provides a solid scientific foundation for developing ASP as a natural anti-tumor agent but also exemplifies a modern research paradigm for investigating complex natural products from “component-structure-function-mechanism” offering a blueprint for mechanistic studies of other herbal medicines (Karanam and Story, 2021; Wang et al., 2023).

The systematic structural characterization of ASP conducted at the outset forms the cornerstone for interpreting its subsequent biological functions. The combined use of techniques such as High-Performance Size Exclusion Chromatography, monosaccharide composition analysis, Scanning Electron Microscopy, and Fourier Transform Infrared Spectroscopy demonstrates the powerful advantage of modern analytical technologies in deciphering the structure of complex natural products. The homogeneous M_W_ profile (55.15 kDa) and single symmetrical peak from HPSEC confirm the high purity of the ASP fraction, excluding interference from other polysaccharides and ensuring the specificity of the biological entity studied (Mislang et al., 2016; Alhanafy et al., 2017; Donato et al., 2019). The high proportions of glucose and galactose in the monosaccharide composition suggest their potential role as backbone components, while the significant content of galacturonic acid endows ASP with polyanionic electrolyte properties, crucial for charge-dependent interactions with biomembranes, proteins, or receptors (Farshidfar et al., 2014; Di Donato et al., 2016; Yachida et al., 2019; Sun et al., 2024). The irregular, porous network structure revealed by SEM not only implies potential good water retention and adsorption capacity but, more importantly, suggests that its large specific surface area could significantly enhance physical interactions with gut microbiota, immune cells, or the intestinal mucosa, providing a structural clue for explaining its prebiotic and immunomodulatory activities *in vivo*. The coexistence of both α- and β-glycosidic linkages, a key finding from FT-IR spectroscopy, is critical evidence of ASP’s structural complexity. This heterotypic linkage configuration suggests a non-single synthase biosynthetic pathway and implies that ASP could be degraded by various microbial glycosidases or bind to multiple immune pattern recognition receptors, thereby eliciting broad and complex biological effects (Yuan et al., 2022).

Histopathological analysis revealed morphological changes consistent with tumor tissue damage following ASP treatment. Observations of vacuolization, inflammatory infiltration, and necrosis are in line with an anti-tumor effect. Notably, we observed the ASPM showing the most pronounced activity-a pattern often associated with biological response modifiers. This suggests that ASP is not merely a cytotoxic agent; its efficacy depends on the host’s responsive state, where excessively high doses might trigger feedback inhibition. In terms of immunomodulation, this study moves beyond the vague description of enhancing immunity to precisely reveal ASP’s “normalizing” effect on the aberrant immune state induced by tumors. It reversed tumor-associated hypertrophy of immune organs, elevated suppressed IFN-γ levels (activating anti-tumor Th1 immunity), and normalized abnormally high levels of IL-2 and TNF-α (alleviating immune dysregulation). This indicates that ASP can intelligently “recalibrate” the body’s immune balance, shifting it from a pro-tumor to an anti-tumor microenvironment. Compared to the purely immunosuppressive effect of the chemotherapeutic agent 5-FU, this multi-directional immunomodulatory capacity highlights the unique advantage and potential of natural polysaccharides in cancer immunotherapy (Zhao et al., 2007; Ecker et al., 2021; Schmitt and Greten, 2021).

The application of untargeted metabolomics allowed this study to uncover the characteristic metabolic disturbances in CRC and the intervention targets of ASP at a systems level. The strength of this approach lies in its hypothesis-free nature, enabling the unbiased discovery of key perturbed pathways, including glycerophospholipid and tryptophan metabolism. The study did not stop at identifying differential metabolites; by correlating the 19 potential biomarkers with pathological indicators like tumor weight and immune parameters, it further refined the list to 12 core metabolites highly relevant to the phenotype, significantly enhancing the biological relevance of the findings. As major membrane components and vital signaling molecules, disturbances in LysoPCs and PCs impact membrane integrity, cell communication, and inflammatory signaling. Meanwhile, we speculate that the changes in the content of tryptophan metabolites (such as L-tryptophan, 5-hydroxyindoleacetic acid, etc.) may link the regulatory effect of ASP with the intestinal microbiota, etc., because tryptophan metabolism is an important bridge connecting the intestinal microecology and the host immunity. Metabolomics thus reveals that ASP’s anti-CRC effect is not merely “cancer cell killing”, but a “correction” of the aberrant metabolic microenvironment upon which tumors thrive (Pierre et al., 2013).

16S rDNA sequencing analysis may reveal a potential link between ASP’s anti-CRC action and the remodeling of the gut microbiota. Through α and β diversity analyses, the study showed that ASP administration was associated with a reversal of the CRC-induced decrease in microbial species richness and a shift in the overall community structure towards a healthier state. At the genus level, ASP treatment was correlated with the suppression of pro-inflammatory genera like *Bacteroides* and the promotion of beneficial bacteria such as Lachnospiraceae_NK4A136_group, which are important producers of SCFAs with known anti-inflammatory and anti-tumor properties. SCFAs (such as butyric acid and propionic acid) not only provide energy for intestinal epithelial cells, but also directly or indirectly exert anti-inflammatory and anti-tumor effects. Therefore, the microbiota remodeling induced by ASP may inhibit intestinal inflammation and tumor progression by promoting the generation of SCFAs, which provides a reasonable explanatory dimension for its anti-CRC mechanism. However, it must be pointed out that the microbiota changes observed in this study are only correlated with the anti-tumor effect of ASP. As no microbiota dependence experiments (such as antibiotic clearance, fecal microbiota transplantation or the use of germ-free mice) have been conducted, it is currently impossible to prove that the remodeling of the intestinal microbiota is a necessary mechanism for ASP to exert anti-tumor effects. Therefore, the regulation of the intestinal microbiota by ASP may be synergistic or partially related to its anti-CRC effect, but its necessity remains to be further verified.

**FIGURE 8:**
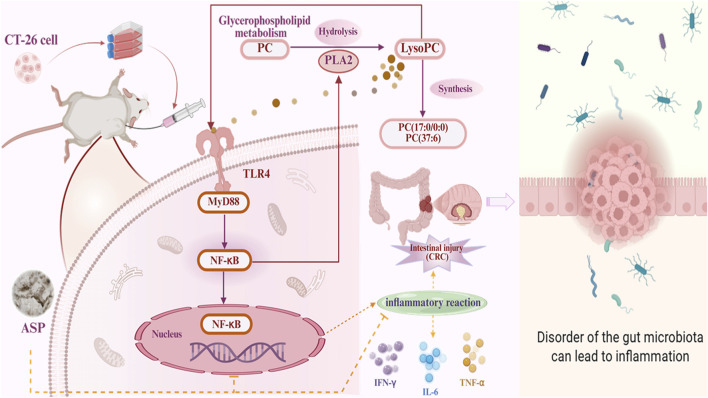
Potential mechanisms of ASP on CRC. (https://www.biorender.com/).

The most outstanding innovation of this study is its integrative approach, which did not treat the microbiota, metabolism, and immunity as isolated systems. Through Spearman correlation network analysis, we constructed a correlative interactive network linking “microbiota-metabolism-immunity-pathology”. This network provides a data-supported framework consistent with the “gut-axis” theory in cancer biology. Subsequently, molecular docking provided a predictive model suggesting potential high-affinity binding between ASP-modulated metabolites and the TLR4 receptor, offering a plausible molecular hypothesis for how microbial signals might be translated into immune modulation. Western blot experiments then demonstrated that ASP treatment is associated with downregulation of key proteins in the PLA2/TLR4/MyD88/NF-kB signaling pathway. This observation result is consistent with the inhibitory effect produced by ASP regulating the metabolite L-tryptophan, though the activation state may reflect broader physiological changes downstream of ASP’s multi-targeted effects, rather than solely a direct ligand-receptor interaction. It is particularly noteworthy that PLA2 is a key enzyme generating LysoPC, which is a potential ligand for TLR4. This implies that ASP might suppress the overactivation of the TLR4 pathway by inhibiting PLA2 and reducing LysoPC production, forming a precise regulatory feedback loop (Buhmeida et al., 2009; Pal et al., 2012; Lancaster et al., 2018) (Figure 8).

It should be noted that the mechanism components expounded in this study, especially the microbiota-metabolite-TLR4 axis, are mainly based on correlation observations and have not yet been verified for causality. Due to the lack of key experiments such as microbial depletion, replenishment rescue experiments or specific pathway inhibitors (such as TLR4 inhibitors), we are currently unable to determine that the remodeling of the gut microbiota is necessary for the anti-tumor effect of ASP. The data from this study support a model where ASP may exert systemic effects by optimizing the intestinal microbiota ecology, but the causal verification of this mechanism still awaits future research. It is important to acknowledge that these *in vivo* findings of study were derived from a single syngeneic CT-26 subcutaneous tumor model. While this model is widely accepted for preliminary evaluation of anti-CRC therapeutics and has provided valuable insights into the multi-omics mechanisms of ASP, it does not fully recapitulate the complexity of the human colonic tumor microenvironment. Future studies could aim to validate these findings in more clinically relevant models, such as orthotopic CRC models, inflammation-driven CRC models (e.g., AOM/DSS), or using additional CRC cell lines, to further strengthen the translational relevance of ASP as a potential anti-CRC agent.

This study found that ASP exhibited significant anti-tumor activity in the CT-26 tumor-bearing mouse model, and its immunomodulatory effect was complementary to traditional chemotherapy drugs. However, this study has not yet explored the combined efficacy of ASP and commonly used chemotherapy drugs in clinical practice (such as 5-FU). In clinical practice, natural products are often used as adjunct treatment methods, combined with chemotherapy drugs to enhance anti-tumor effects and reduce toxic and side effects (Liang et al., 2021). For instance, although 5-FU has a strong tumor-killing ability, the immunosuppression and bone marrow toxicity it causes limit its long-term application (Lu et al., 2015). As a polysaccharide with immune regulation and metabolic remodeling functions, ASP may theoretically enhance the therapeutic effect of 5-FU through a synergistic mechanism and alleviate its immunosuppression effect at the same time. Future studies can design a combined treatment regimen of “ASP plus low-dose 5-FU” to systematically evaluate the effects of combined medication on tumor suppression rate, immune organ index, cytokine spectrum and intestinal microbiota-metabolic axis. If the combined therapy shows a synergistic anti-tumor effect and can reduce 5-FU immune damage, it will provide an important basis for the clinical transformation of ASP as an adjuvant therapeutic agent for CRC and further enhance its application potential.

In summary, this study not only elucidates the anti-CRC mechanism of ASP but, more importantly, presents a comprehensive research strategy that spans from macroscopic phenotypes to microscopic molecules, encompassing both the host and its symbiotic microbes, as well as chemical structures and biological functions. It compellingly illustrates that the anti-tumor activity of natural polysaccharides is a networked process initiated by the microbiota, mediated by metabolites, and executed through immune and signaling pathways-a multi-component and synergistic endeavor. This offers a novel perspective along with potential targets for future development of tumor intervention strategies grounded in microecological regulation and metabolic reprogramming.

## 5 Conclusion

In conclusion, our integrated study systematically investigates ASP as a well-defined acidic heteropolysaccharide with certain anti-CRC efficacy. This remodeling of the microbial community and increase in the content of some SCFAs may be associated with a systemic shift in host metabolism, specifically towards normalization in the dysregulated glycerophospholipid and tryptophan pathways. Then leading to the suppression of the TLR4/MyD88/NF-κB signaling pathway. Ultimately, ASP orchestrates a concerted biological response encompassing reversal of metabolic abnormalities, and multi-faceted immunomodulation to reinstate anti-tumor immunity. Our findings not only provide a comprehensive mechanistic basis for the application of ASP against CRC but also establish a robust “microbiota-metabolism-immunity” framework for future development of natural polysaccharides as multi-targeted therapeutic or adjuvant agents.

## Data Availability

The original contributions presented in the study are publicly available. This data can be found here: https://figshare.com/s/a0339da3c0c932633553, DOI: https://doi.org/10.6084/m9.figshare.31274020.
