# Supplementary material for: Unraveling the anti-colorectal cancer mechanisms of Acanthopanax senticosus polysaccharide: a multi-omics investigation into gut microbiota-metabolism-immunity crosstalk
Source: Front Pharmacol. 2026 Feb 24;17:1749532. doi: 10.3389/fphar.2026.1749532 (PMC12971678; doi:10.3389/fphar.2026.1749532)
Supplement: Supplementary file 1 [file Supplementaryfile1.docx]

Supplementary Material

# Supplementary Figures


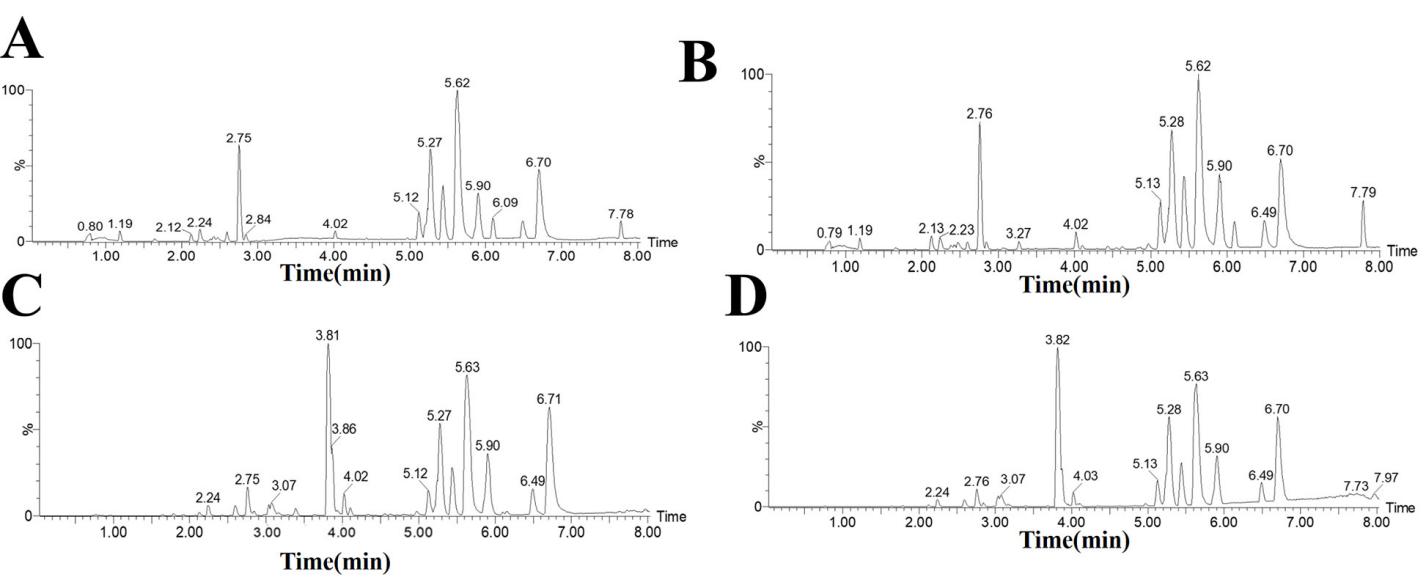


**Supplementary Figure 1.** BPI chromatogram of blood metabolism between C group and M group in positive mode and negative mode.

(A) Control group in positive mode; (B) Control group in negative mode; (C) Model group in positive mode. (D) Model group in negative mode.

# Supplementary Tables

Table S1. Identification results of blood biomarkers in CRC tumor-bearing mice models

| No. | Rt/min | M/Z | Adduct ion | Formula | Metabolite name | Fragments | Trend |
| --- | --- | --- | --- | --- | --- | --- | --- |
| 1 | 1.91 | 164.0722 | [M-H]^-^ | C_9_H_11_NO_2_ | Dl-Phenylalanine | 164.0722; 147.0427 | ↓** |
| 2 | 1.91 | 192.0663 | [M+H]^+^ | C_10_H_9_NO_3_ | 5-hydroxyindoleacetic acid | 192.0663; 146.0610 | ↓** |
| 3 | 1.92 | 118.0645 | [M+H]^+^ | C_8_H_7_N | Indole | 118.0645; 91.0542 | ↓** |
| 4 | 2.13 | 132.0803 | [M+H]^+^ | C_9_H_9_N | 3-Methylindole | 132.0803; 116.7792 | ↓** |
| 5 | 2.13 | 203.0836 | [M-H]^-^ | C_11_H_12_N_2_O_2_ | L-tryptophan | 203.0836; 185.8766 | ↓** |
| 6 | 2.32 | 162.0547 | [M+H]^+^ | C_9_H_7_NO_2_ | Indole-3-carboxylic acid | 162.0547; 146.0358 | ↓* |
| 7 | 2.76 | 100.1117 | [M+H]^+^ | C_6_H_13_N | Cyclohexylamine | 100.1117; 84.0801 | ↑** |
| 8 | 2.9 | 158.0609 | [M-H]^-^ | C_10_H_9_NO | Indole-3-acetaldehyde | 158.0609; 130.0656 | ↑** |
| 9 | 3.81 | 254.9952 | [M-H]^-^ | C_10_H_8_O_6_S | 4-Methylumbelliferone sulfate | 254.9952; 175.0410 | ↓** |
| 10 | 4.18 | 301.0023 | [M-H]^-^ | C_14_H_6_O_8_ | Ellagic acid | 301.0023; 256.9874 | ↓** |
| 11 | 5.13 | 568.3413 | [M+H]^+^ | C_30_H_50_NO_7_P | LysoPC(22:6) | 568.3413; 385.2737; 164.665 | ↓* |
| 12 | 5.65 | 518.3239 | [M+H]^+^ | C_26_H_48_NO_7_P | LysoPC(18:3(6Z,9Z,12Z)/0:0) | 518.3239; 173.4123; 104.1070 | ↓* |
| 13 | 5.72 | 544.3419 | [M+H]^+^ | C_28_H_50_NO_7_P | LysoPC(20:4/0:0) | 544.3419; 526.3292; 184.0733 | ↓** |
| 14 | 5.78 | 790.5465 | [M-H]^-^ | C_45_H_78_NO_8_P | PC(37:6) | 790.5465; 597.4888; 241.2173 | ↓** |
| 15 | 5.94 | 735.0234 | [M-H]^-^ | C_40_H_80_NO_8_P | PE(20:3(8Z,11Z,14Z)/20:3(8Z,11Z,14Z)) | 735.0234; 478.9663; 184.2468 | ↓** |
| 16 | 6.49 | 508.3405 | [M-H]^-^ | C_25_H_52_NO_7_P | PC(17:0/0:0) | 508.3405; 327.2899; 184.0726 | ↓* |
| 17 | 7.47 | 820.6091 | [M+H]^+^ | C_44_H_86_NO_10_P | PS(18:0/20:0) | 820.6091; 635.5973 | ↑** |
| 18 | 7.47 | 282.2801 | [M+H]^+^ | C_18_H_35_NO | Oleamide | 282.2801; 247.5066 | ↓** |
| 19 | 7.5 | 818.6080 | [M+H]^+^ | C_48_H_84_NO_7_P | PC(40:2) | 818.608; 538.5066; 323.5066 | ↑** |

Note: ↑: The content of blood biomarkers increased in CRC model mice; ↓: The content of blood biomarkers decreased in CRC model mice. *P<0.05, **P<0.01, C group vs M group.

Table S2. Analysis of metabolic pathways regulated by ASP therapy for CRC.

| **Pathway name** | **Hits** | **Total** | **-Log p** | **Impact** |
| --- | --- | --- | --- | --- |
| Glycerophospholipid metabolism | 4 | 36 | 5.1144 | 0.26332 |
| Tryptophan metabolism | 3 | 41 | 3.2728 | 0.17085 |
| Linoleic acid metabolism | 1 | 5 | 1.6565 | 0.00000 |
| alpha-Linolenic acid metabolism | 1 | 13 | 1.2482 | 0.00000 |
| GPI-anchor biosynthesis | 1 | 15 | 1.1877 | 0.00639 |
| Arachidonic acid metabolism | 1 | 44 | 0.7442 | 0.00000 |

Table S3. Molecular docking results of 4 compounds with TLR4, MyD88, and NF-κB

| **Components** | **LibDock Score** | | |
| --- | --- | --- | --- |
|  | **TLR4** | **MyD88** | **NF-κB** |
| LysoPC(22:6) | -6.2 | -6.8 | -7.7 |
| PC(17:0/0:0) | -5.9 | -7.2 | -7.5 |
| L-tryptophan | -6.4 | -6.7 | -7.2 |
| PC(37:6) | -9.6 | -7.9 | -8.3 |
